# IUC26589-95 First-Line or Maintenance Immune Checkpoint Inhibitors in Older Patients with Advanced Urothelial Carcinoma: A Systematic Review and Meta-Analysis

**DOI:** 10.1093/oncolo/oyag286.004

**Published:** 2026-08-21

**Authors:** D Incognito, A D'arienzo, C Barraco, A Nicastro, S Facchini, G Facchini, D Incognito

**Affiliations:** PhD Student in Translational Molecular Medicine and Surgery, Department of Clinical and Experimental Medicine, University of Messina - Italy; Oncology Unit, S. Maria Delle Grazie Hospital, Pozzuoli, Naples - Italy; Oncology Unit, S. Maria Delle Grazie Hospital, Pozzuoli, Naples - Italy; Oncology Unit, S. Maria Delle Grazie Hospital, Pozzuoli, Naples - Italy; Experimental Clinical Abdominal Oncology Unit, Istituto Nazionale Tumori- IRCCS-Fondazione G. Pascale, Naples - Italy; Oncology Unit, S. Maria Delle Grazie Hospital, Pozzuoli, Naples - Italy; Bronte - Italy

## Abstract

**Background:**

Evidence on immune-based treatment in older patients with advanced urothelial carcinoma are limited because age-specific outcomes are usually exploratory. We assessed the efficacy of first-line and maintenance immune-based strategies in patients aged ≥65 years.

**Methods:**

PubMed/MEDLINE, Embase, Cochrane and major oncology congresses were searched from Jan 2015 to May 2026 for randomized trials reporting age-specific hazard ratios (HRs) for overall survival (OS) or progression-free survival (PFS). The primary analysis pooled first-line combination regimens versus platinum-based chemotherapy using random-effects models. For maintenance treatment, an anchored Bucher indirect comparison used avelumab as the shared treatment node, evaluating PFS as the primary endpoint.

**Results:**

Six first-line randomized trials of first-line treatment combination were identified. A number of 2,678 patients aged ≥65 years were represented in experimental and control groups. All first-line estimates came from exploratory age subgroup analyses. Combination first-line regimens improved OS versus chemotherapy (HR 0.79, 95% CI 0.64–0.96), although heterogeneity was substantial (I² = 69.8%; p  =  0.0029). Pooled estimates were HR 0.56 (95% CI 0.26–1.20) for antibody-drug conjugate plus immunotherapy (ADC+IO), HR 0.86 (95% CI 0.64–1.14) for ICI plus chemotherapy, and HR 0.91 (95% CI 0.35–2.38) for dual ICI combinations. Treatment effects differed across categories (p < 0.0001), but comparisons were confounded by differences in population selection, cisplatin eligibility, and trial design. ICI monotherapy did not show an OS benefit: pembrolizumab versus chemotherapy yielded HR 1.15 (95% CI 0.84–1.59), while the atezolizumab estimate was HR 0.98 (95% CI 0.79–1.21). In maintenance, avelumab improved PFS versus best supportive care (HR 0.46, 95% CI 0.37–0.57). The indirect comparison estimated an HR of 0.31 (95% CI 0.18–0.54; p < 0.0001) for avelumab plus sacituzumab govitecan versus best supportive care.

**Conclusions:**

In clinically fit older patients, combination-based first-line and maintenance treatment was associated with improved OS and PFS. In contrast, the available data did not show a survival benefit with ICI monotherapy. These findings argue against routine de-escalation strategies on the basis of age alone. Further age-specific efficacy, safety, and geriatric assessment data are needed to guide treatment selection.

**IUC26589-95 Figure 1:**
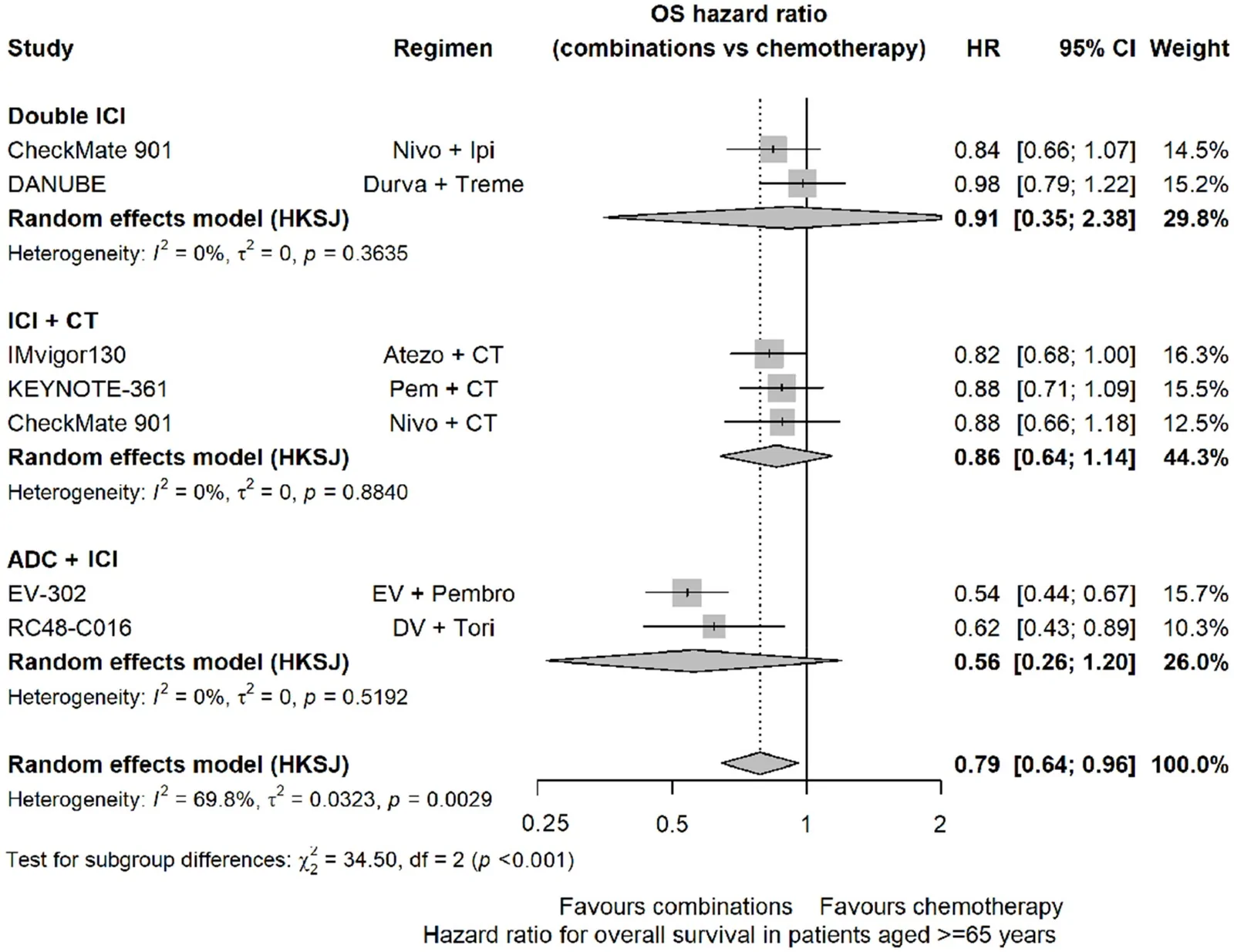
Pooled OS HR for fist-line treatment combinations

